# Triglyceride–glucose index as a prognostic marker of endovascular thrombectomy in patients with ischemic stroke: a retrospective study

**DOI:** 10.3389/fmed.2025.1640543

**Published:** 2025-09-30

**Authors:** Zhaoliang Sun, Xuchen Meng, Zixian Mei, Tanjun Deng, Xin Lv, Jiexi Xiao, Lin Zhu, Dingzhong Tang, Weijie Zhong, Yi Li

**Affiliations:** ^1^Department of Neurosurgery, Ninth People’s Hospital Affliated to Shanghai Jiao Tong University School of Medicine, Shanghai, China; ^2^Department of Neurology Medical, Jinshan Branch of Shanghai Sixth People’s Hospital, Shanghai, China

**Keywords:** acute ischemic stroke, endovascular thrombectomy, triglyceride-glucose index, prognosis, prognostic marker

## Abstract

**Objective:**

This study investigates the association between the triglyceride–glucose (TyG) index, a surrogate marker for insulin resistance, and clinical outcomes in patients with acute ischemic stroke (AIS) after endovascular thrombectomy (EVT).

**Methods:**

This retrospective study included 179 patients with AIS who underwent EVT. The TyG index was calculated as ln[triglycerides (TGs) (mg/dL) × glucose (mg/dL)/2] from admission blood samples. The primary outcome was functional status at 90 days post-stroke, assessed by the modified Rankin Scale (mRS). A multivariate logistic regression analysis was performed to evaluate the association between the TyG index and outcomes after adjusting for potential confounders.

**Results:**

Among the 179 patients, 77 (43.0%) had favorable outcomes (mRS ≤ 2) and 102 (57.0%) had poor outcomes (mRS > 2) at 90 days post-stroke. The TyG index was significantly higher in the poor outcome group compared to the favorable outcome group. The receiver operating characteristic curve analysis showed that the TyG index (area under the ROC curve, AUC = 0.714) had superior predictive value compared with either glucose (AUC = 0.618) or TGs (AUC = 0.574) alone or their combination (AUC = 0.633). The optimal cut-off value for the TyG index was 8.795, with a sensitivity of 0.569 and a specificity of 0.753. A multivariate logistic regression analysis confirmed that the TyG index was independently associated with poor outcomes after adjusting for conventional prognostic factors. Adding the TyG index to a prediction model significantly improved its performance (AUC from 0.776 to 0.826, *p* = 0.032). Subgroup analyses revealed that the TyG index had enhanced prognostic value in elderly (≥65 years, AUC = 0.747) and male patients (AUC = 0.726).

**Conclusion:**

Elevated TyG index is independently associated with poor outcomes in patients with AIS after EVT. The TyG index demonstrates superior predictive performance compared to individual metabolic parameters and significantly improves outcome prediction when added to conventional prognostic factors. These findings suggest that the TyG index may serve as a valuable prognostic marker for risk stratification in patients with AIS undergoing EVT.

## Introduction

Acute ischemic stroke (AIS) remains a leading cause of disability and mortality worldwide despite advances in treatment strategies (1). Endovascular thrombectomy (EVT) has revolutionized the management of AIS with large vessel occlusion (LVO), significantly improving functional outcomes compared to medical therapy alone (2, 3). However, a substantial proportion of patients still experience poor outcomes after EVT, highlighting the need for reliable prognostic biomarkers to identify high-risk patients who may benefit from more intensive monitoring and management (4).

Current prediction models for the prognosis of patients with AIS undergoing EVT mainly rely on age, National Institutes of Health Stroke Scale (NIHSS), hypertension (5), and imaging data (6), with relatively few metabolic factors in prognosis prediction (7). Notably, metabolic disorders play a pivotal role in the pathophysiology of cerebral ischemia–reperfusion injury, contributing to endothelial dysfunction, inflammation, and thrombosis (8). Therefore, compared to static indicators (e.g., NIHSS scores), metabolic parameters can reflect cerebral tissue metabolic status and provide dynamic prognostic assessment, potentially improving prognostic accuracy.

The triglyceride–glucose (TyG) index, calculated from TG and glucose levels, has emerged as a simple and reliable surrogate marker for IR (9), showing strong correlation with the cardiovascular and cerebrovascular outcomes. Recent studies have demonstrated that an elevated TyG index correlates with an increased risk of coronary artery disease and carotid atherosclerosis (10–12). In the context of stroke, a few studies have explored the relationship between TyG index and outcomes in patients with AIS treated with intravenous thrombolysis; others report an association between elevated TyG index and mortality rate (13, 14). However, the prognostic value of the TyG index in patients with AIS undergoing EVT remains largely unexplored.

Therefore, this single-center retrospective study aimed to investigate the association between the TyG index and clinical outcomes in patients with AIS after EVT. The findings of this study may provide insights into the prognostic value of the TyG index in patients with AIS undergoing EVT and potentially inform risk stratification and personalized management strategies.

## Methods

### Study design and participants

**Figure 1:**
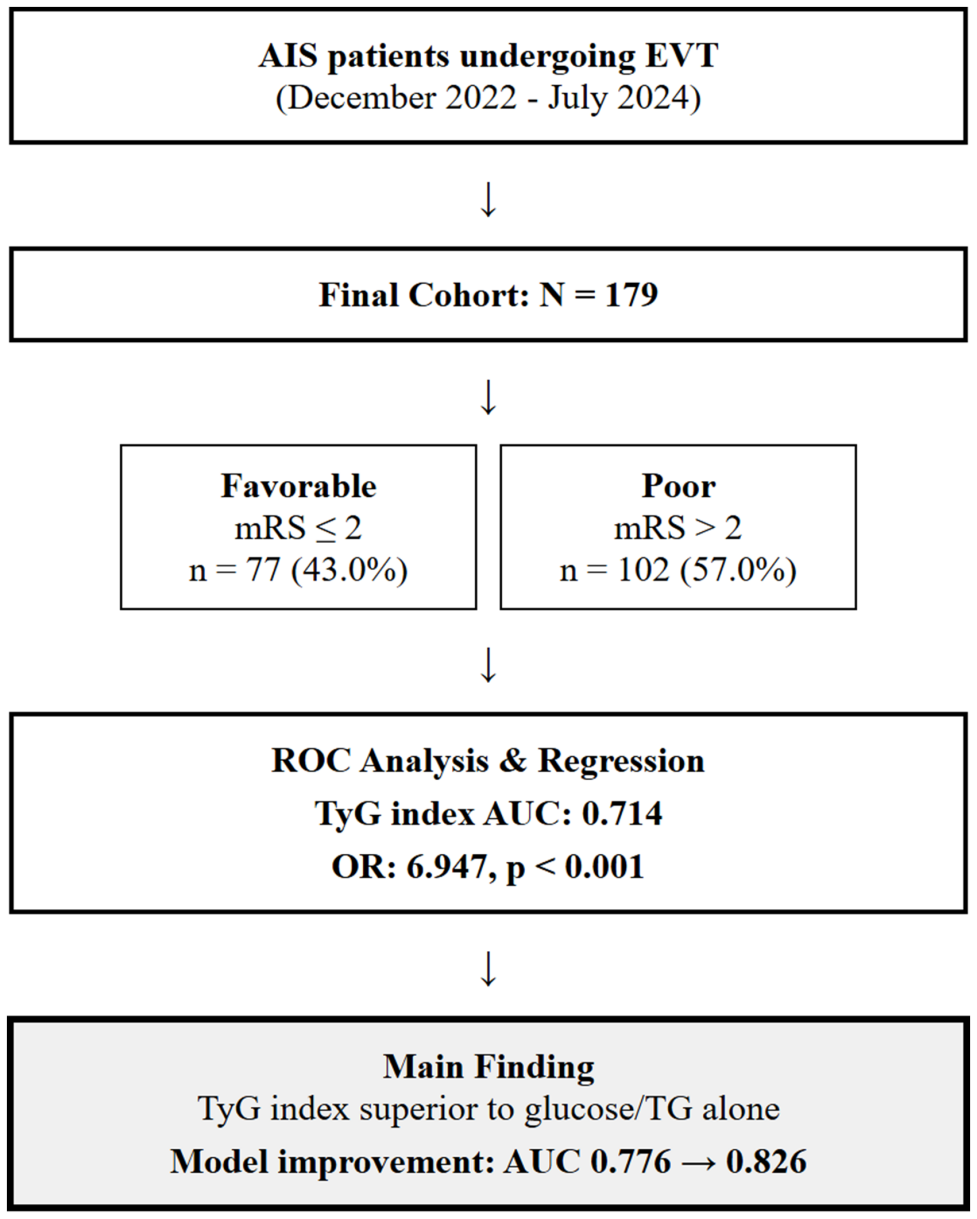
A flowchart of the inclusion of the study cohort. AIS, acute ischemic stroke; EVT, endovascular thrombectomy; TyG, triglyceride-glucose; mRS, modified Rankin Scale; AUC, area under curve.

The study protocol was approved by the Ethics Committee of Shanghai Ninth People’s Hospital, Shanghai Jiao Tong University School of Medicine (approval number: SH9H-2024-T347-1), and the requirement for informed consent was waived, given the retrospective nature of the study and the use of anonymized data. We included consecutive adult patients with AIS who underwent EVT between December 2022 and July 2024. The inclusion criteria were as follows: (1) anterior circulation ischemic stroke confirmed by CT or MRI; (2) age 18 years or older; (3) symptom onset to admission ≤24 h; (4) thrombectomy performed; (5) follow-up for at least 90 days; and (6) complete baseline laboratory data, such as TGs and glucose levels. Exclusion criteria included (1) incomplete clinical and laboratory data, (2) missing TyG index or prognostic data, and (3) other complications or fatal diseases, combined with malignant tumors, active infection, end-stage liver and kidney insufficiency, and expected survival < 3 months. A flowchart of the inclusion of the study cohort is shown in Figure 1. The sample size was determined by convenience sampling based on all eligible patients who met the inclusion and exclusion criteria during the study period rather than by prospective power calculation. Post-hoc power analysis confirmed that our sample size of 179 patients provided approximately 80% power to detect the observed differences in TyG index between outcome groups (*α* = 0.05).

### Data collection and definitions

Demographic and clinical data were collected from electronic medical records, including age, sex, history of comorbidities, vascular risk factors (hypertension, smoking status, and alcohol consumption), infarction hemisphere, modified Thrombolysis in Cerebral Infarction (mTICI), and TyG. Stroke severity was assessed using the NIHSS at admission. Level of consciousness was evaluated using the Glasgow Coma Scale (GCS).

1. Imaging data collection and assessment: (1) Neuroimaging protocol: All patients underwent standardized neuroimaging evaluation according to institutional protocols. Initial imaging included non-contrast computed tomography (NCCT) of the head, computed tomography angiography (CTA) of the head and neck, and computed tomography perfusion (CTP) when clinically indicated (15).
2. Vessel occlusion assessment: Large vessel occlusion was identified and characterized using CTA. Occlusion sites were classified according to established anatomical criteria: internal carotid artery (ICA) occlusion, such as terminal ICA and ICA-T occlusions, and middle cerebral artery (MCA) occlusion (3).
3. Midline shift measurement: Midline shift was measured on NCCT as the maximum perpendicular distance between the actual midline and the expected midline. Measurements were categorized as 0 mm (no shift) or >5 mm (significant shift) based on established thresholds associated with clinical outcomes.
4. Reperfusion assessment: mTICI scores were categorized as 0 (no perfusion), 1 (penetration with minimal perfusion), 2a (partial filling <50% of territory), 2b (partial filling 50–99% of territory), 2c (near-complete perfusion with slow flow), and 3 (complete perfusion). For outcome analysis, successful reperfusion was defined as mTICI 2b, 2c, or 3 (16).

Laboratory data collected included glucose and TG levels obtained from the first blood sample upon hospital admission, specifically collected prior to any therapeutic intervention, such as EVT. The TyG index was calculated as ln[TGs (mg/dL) × glucose (mg/dL)/2] using these pre-treatment admission values to ensure reflection of baseline metabolic status. Blood samples were collected immediately upon patient arrival to the emergency department as part of routine acute stroke evaluation protocols, typically within the first hour of hospital presentation (17). However, we acknowledge a fundamental limitation: our study did not distinguish between chronic insulin resistance and stress-induced hyperglycemia (SIH). We lacked HbA1c data to calculate stress hyperglycemia ratios, preventing differentiation between patients with chronic metabolic dysfunction versus those with normal baseline glucose tolerance who developed transient hyperglycemia due to stroke-induced neuroendocrine activation. This represents a critical confounding factor as these two conditions have entirely different pathophysiological mechanisms and prognostic implications. We selected the TyG index over other insulin resistance markers based on several considerations: (1) our preliminary analysis showed that TG/high-density lipoprotein (HDL)-C ratio, while statistically significant (*p* = 0.023), had limited predictive capacity (area under the ROC curve; AUC = 0.607); (2) derivative indices such as TyG-(body mass index)BMI and TyG-waist circumference (WC) could not be calculated due to incomplete anthropometric data collection in our retrospective design; and (3) the TyG index demonstrates superior predictive performance compared to individual metabolic parameters and is readily available from routine laboratory tests in emergency settings. All biochemical analyses were performed in the central laboratories of each participating center according to standard methods.

### Outcome measures

The primary outcome was functional status at 90 days post-stroke, as measured by the modified Rankin Scale (mRS). A favorable outcome was defined as an mRS score of ≤2, indicating functional independence. Poor outcome was defined as an mRS score of >2, indicating moderate to severe disability or death (18). Assessment of mRS was performed by trained neurologists who were blinded to patients’ baseline data either through in-person follow-up visits or structured telephone interviews with patients or their caregivers.

### Statistical analysis

Continuous variables were presented as mean ± standard deviation or median (interquartile range) depending on their distribution, and categorical variables were presented as frequencies and percentages. For between-group comparisons, we used the independent *t*-test or Mann–Whitney U test for continuous variables and the Chi-square test or Fisher’s exact test for categorical variables, as appropriate.

The receiver operating characteristic (ROC) curve analysis was performed to determine the optimal cut-off value of the TyG index for predicting poor outcomes after EVT. The optimal cut-off value was determined using the Youden index method (J = sensitivity + specificity − 1), which identifies the threshold that maximizes the sum of sensitivity and specificity, thus providing the best discrimination between patients with favorable and poor outcomes. This approach ensures an optimal balance between true positive and true negative rates while minimizing both false positive and false negative classifications. The area under the ROC curve AUC was used to evaluate the reliability of the prediction models. The multivariable forward stepwise logistic regression analysis was used to assess the associations between clinical characteristics. Given our sample size of 179 patients with 102 poor outcome events, we followed the guideline of requiring at least 10 events per variable to avoid overfitting (19). Variables for inclusion in the multivariate logistic regression model were selected based on a two-step approach: (1) variables with a *p*-value of <0.05 in univariate analysis were considered as candidates and (2) final variable selection was based on both statistical significance and clinical relevance established in previous stroke outcome studies (6). The selected variables included onset symptoms, hypertension, midline shift, admission NIHSS score, and TyG index. This approach ensures adequate statistical power while maintaining clinical interpretability (20).

Subgroup analyses were conducted to evaluate the predictive value of the TyG index in different age groups (<65 years vs. ≥65 years) and by sex (male vs. female). For each subgroup, ROC curve analyses were performed to calculate the AUC for the TyG index. Statistical analyses were performed using R software version 4.0.3 (R Foundation for Statistical Computing, Vienna, Austria). A two-tailed *p*-value of < 0.05 was considered statistically significant.

## Results

### Baseline characteristics

A total of 179 patients with AIS who underwent thrombectomy were included in this analysis. Among them, 77 patients (43.0%) had favorable outcomes and 102 patients (57.0%) had poor outcomes. When comparing the baseline characteristics between these two groups, we observed significant differences in several parameters (Table 1). The proportion of hypertension was significantly higher in the poor outcome group compared to the favorable outcome group (44.1% vs. 25.7%, *p* = 0.011). Similarly, diabetes mellitus was more common in the poor outcome group (19.0% vs. 6.1%, *p* = 0.004). The median GCS score on admission was significantly lower in the poor outcome group compared to the favorable outcome group [8 (6–13) vs. 12 (9–14), *p* < 0.001], while the median NIHSS score was significantly higher [17 (10–24) vs. 12 (6–18), *p* = 0.001]. Regarding metabolic parameters, the mean TyG index was significantly higher in the poor outcome group (8.55 vs. 8.87, *p* < 0.0001). Similarly, both glucose (159.03 vs. 131.25 mg/dL, *p* = 0.004) and TG (114.7 vs. 90.28 mg/dL, *p* = 0.011) were significantly elevated in the poor outcome group.

**Table 1:** Baseline clinical characteristics of patients with favorable and unfavorable prognosis after EVT for AIS.

Characteristics	Favorable outcome group (*n* = 77)	Poor outcome group (*n* = 102)	*p-*value
Sex, *n* (%)		0.993	
Male	46 (25.7%)	61 (34.1%)	
Female	31 (17.3%)	41 (22.9%)	
Age (year), mean ± sd	70.03 ± 12.44	69.32 ± 13.97	0.728
Symptoms of onset, *n* (%)			0.017
Coma	7 (3.9%)	23 (12.8%)	
Others	70 (39.1%)	79 (44.1%)	
GCS score, mean (IQR)	12 (9–14)	8 (6–13)	<0.001*
History of comorbidities			
Hypertension, *n* (%)	46 (25.7%)	79 (44.1%)	0.011
Atrial fibrillation, n (%)	27 (15.1%)	36 (20.1%)	0.975
Diabetes, *n* (%)	11 (6.1%)	34 (19%)	0.004
Hyperlipoidemia, *n* (%)	2 (1.1%)	3 (1.7%)	1.000
Prior stroke/TIA, *n* (%)	9 (5%)	17 (9.5%)	0.349
Coronary heart disease, *n* (%)	9 (5%)	12 (6.7%)	0.987
Smoke, *n* (%)	9 (5%)	25 (14%)	0.030
Alcohol, *n* (%)	5 (2.8%)	19 (10.6%)	0.018
Infarction hemisphere, *n* (%)			0.118
Left	37 (20.7%)	61 (34.1%)	
Right	40 (22.3%)	41 (22.9%)	
Occlusion artery, *n* (%)			0.645
ICA	22 (12.3%)	26 (14.5%)	
MCA	55 (30.7%)	76 (42.5%)	
Reperfusion, *n* (%)			0.896
mTICI 2b/2c/3	72 (40.2%)	97 (54.2%)	
mTICI 0/1/2a	5 (2.8%)	5 (2.8%)	
TOAST, *n* (%)			0.448
CE	11 (6.1%)	22 (12.3%)	
LAA	31 (17.3%)	36 (20.1%)	
Others	35 (19.6%)	44 (24.6%)	
Mid-Line Shift, *n* (%)			0.005
0 mm	72 (40.2%)	80 (44.7%)	
>5 mm	5 (2.8%)	22 (12.3%)	
Onset to examination time (h), median (IQR)	3.5 (2–8)	5 (3–9)	0.519*
Admission NIHSS, median (IQR)	12 (6–18)	17 (10–24)	0.001*
TG (mg/dL), mean ± sd	90.28 ± 37.15	114.70 ± 85.38	0.011
Glucose (mg/dL), mean ± sd	131.25 ± 45.69	159.03 ± 79.24	0.004
TyG index, mean ± sd	8.55 ± 0.46	8.87 ± 0.65	<0.001

SD, standard deviation; TIA, transient ischemic attack; TOAST, Trial of ORG 10172 in Acute Stroke Treatment; LAA, large-artery atherosclerosis; CE, cardioembolic; mTICI, modified Thrombolysis in Cerebral Infarction; TC, total cholesterol; TG, triglyceride; mRS, modified Rankin Scale; TyG, triglyceride-glucose.

*The Mann–Whitney U test was used for non-normally distributed variables; the independent *t*-test was used for normally distributed variables.

### ROC curve analysis of TyG index, glucose, and TGs

**Figure 2:**
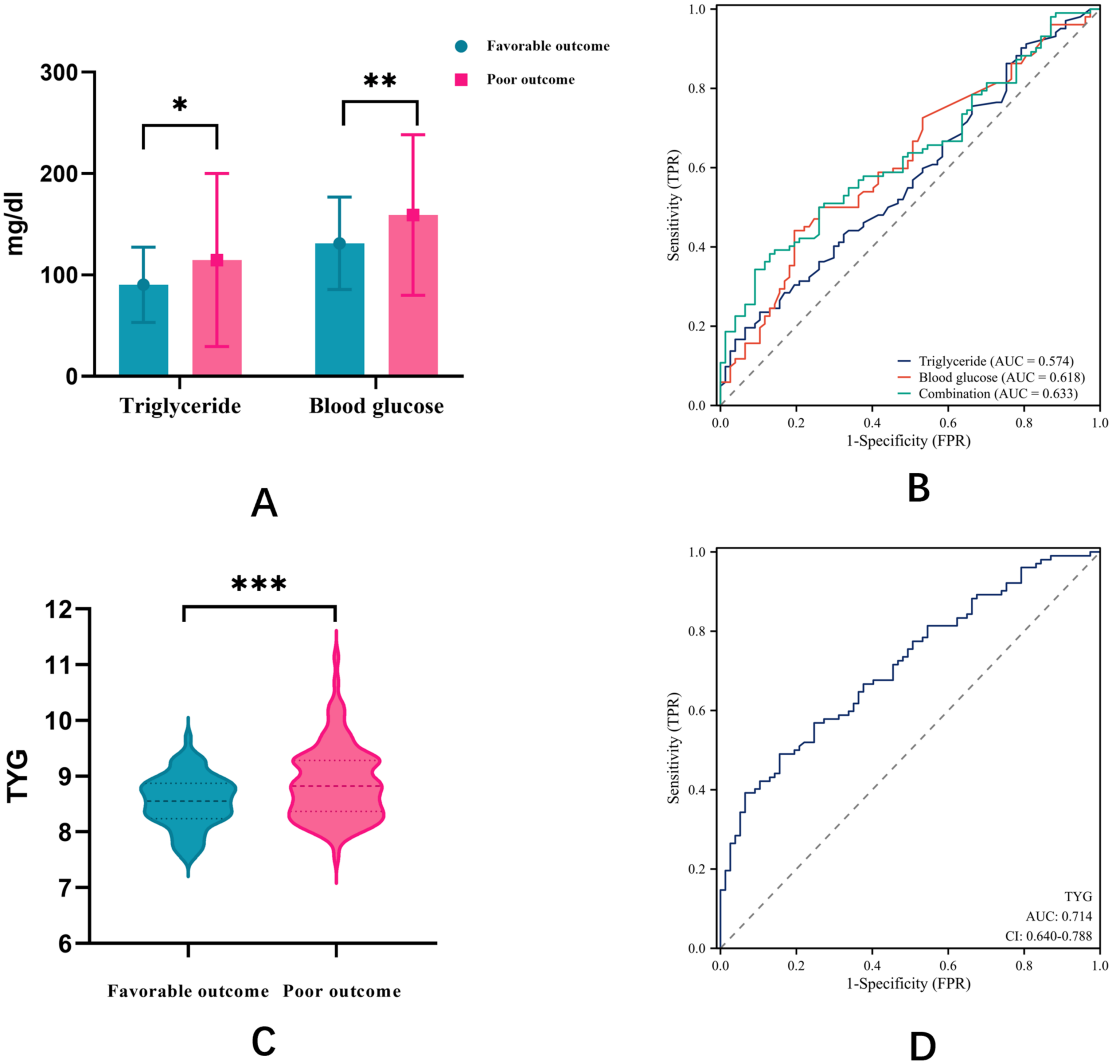
**(A)** Differences between favorable and poor outcome groups for glucose, TGs, and combination; **(B)** ROC curve analysis of glucose, TGs, and combination. **(C)** Differences between favorable and poor outcome groups for TyG index; **(D)** ROC curve analysis of TyG index.

TyG index, glucose, and TGs showed significant differences between the favorable and poor outcome groups for all three parameters. To compare the predictive value of TyG index with that of its individual components for stroke outcomes, we conducted ROC curve analyses (Figure 2). The ROC curve analysis revealed that the AUC for TGs alone was 0.574, that for glucose alone was 0.618, and that for the combination of both parameters was 0.633 (Figure 2B). Notably, the TyG index demonstrated superior predictive performance with an AUC of 0.714 (Figure 2D). The optimal cut-off value for TyG index was determined to be 8.795, with a sensitivity of 0.569 and a specificity of 0.753. These results indicate that the TyG index has better discriminative ability for predicting poor outcomes after EVT compared to either glucose or TGs alone or their simple combination.

### Multivariate logistic regression analysis and model comparison

**Figure 3:**
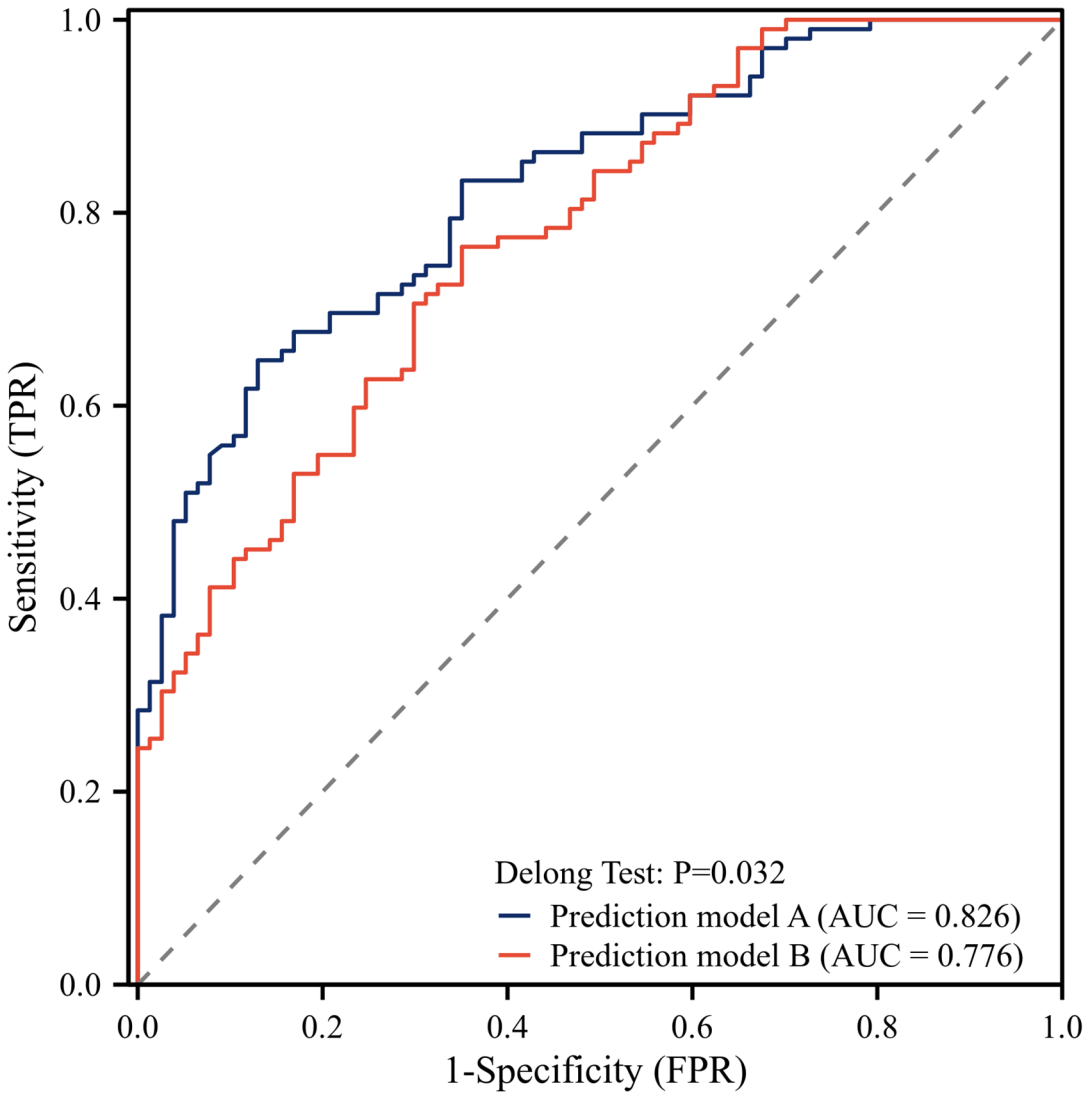
ROC curve analysis of the model. Model A: with TyG index; Model B: without TyG index.

Based on the results of univariate logistic regression analysis, we performed the multivariable logistic regression analysis using forward stepwise selection. The final model included onset symptoms, hypertension, midline shift, admission NIHSS score, and TyG index as independent variables (Table 2). To evaluate the added value of the TyG index in outcome prediction, we compared two prediction models: one with and one without the TyG index. The ROC curve analysis showed that the model including the TyG index had a significantly higher AUC (0.826) compared to the model without TyG index (0.776). The DeLong test confirmed that this difference was statistically significant (*p* = 0.032), suggesting that the addition of the TyG index significantly improved the predictive performance of the model for outcomes after thrombectomy (Figure 3).

**Table 2:** Univariate logistic regression analysis.

Multiple regression analysis	OR	95%CI	*p-*value
Symptom (coma to others)	2.876	1.008–8.208	0.048
Hypertension	2.907	1.274–6.634	0.011
Mid-Line Shift	5.759	1.756–18.883	0.004
Admission NIHSS	1.088	1.037–1.142	0.001
TyG	6.947	3.178–15.184	<0.001

### Subgroup analysis by age and gender

**Figure 4:**
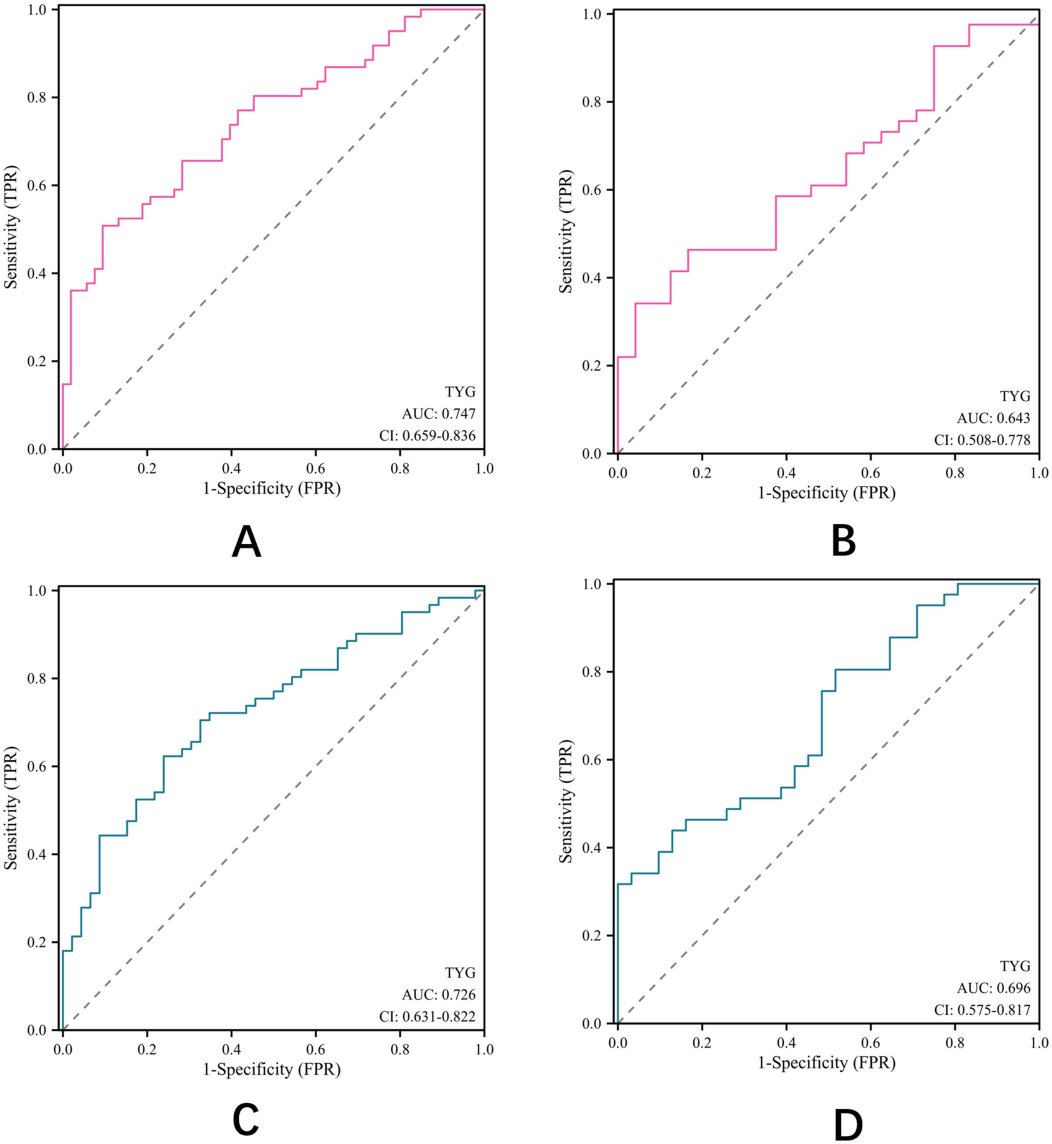
ROC curve analysis of TyG index. **(A)** aged ≥65 years; **(B)** Aged <65; **(C)** male; **(D)** female.

Having established TyG index as a potential effective predictor of outcomes after thrombectomy in patients with AIS, we conducted subgroup analyses to evaluate its predictive value across different age and gender groups (Figure 4). In patients aged ≥65 years (Figure 4A), the TyG index showed a higher predictive value (AUC = 0.747) compared to patients aged <65 years (AUC = 0.643) (Figure 4B). Similarly, the predictive value of TyG index was slightly higher in male patients (AUC = 0.726) (Figure 4C) than in female patients (AUC = 0.696) (Figure 4D). These findings suggest that the TyG index may have enhanced prognostic value in elderly male patients with AIS after EVT.

## Discussion

In this retrospective study, we investigated the relationship between the TyG index and clinical outcomes in patients with AIS after EVT. Our findings demonstrate that an elevated TyG index is independently associated with unfavorable outcomes, with superior predictive value compared to either glucose or TGs alone. Furthermore, the addition of the TyG index to conventional prognostic factors significantly improved the predictive performance of the model, particularly in elderly male patients.

Our choice of the TyG index was based on both empirical evidence and practical considerations. While derivative indices, such as TyG-BMI and TyG-WC, have shown enhanced predictive value in some studies (21), these were not feasible in our retrospective analysis due to incomplete anthropometric data collection. Our preliminary evaluation of the TG/HDL-C ratio, another commonly used insulin resistance marker, revealed limited discriminative ability (AUC = 0.607) despite statistical significance, supporting our decision to use the TyG index, which demonstrated superior predictive performance.

The association between elevated TyG index and poor outcomes after thrombectomy may be explained by several pathophysiological mechanisms, though our inability to distinguish chronic insulin resistance from stress-induced hyperglycemia fundamentally limits mechanistic interpretation. For patients with true chronic insulin resistance, mechanisms may include endothelial dysfunction, impaired collateral circulation, and enhanced atherothrombosis. However, for patients with stress-induced hyperglycemia, the elevated TyG index may simply reflect stroke severity through neuroendocrine activation rather than representing an independent pathophysiological pathway. The potential mechanisms include the following: first, insulin resistance, as reflected by high TyG index, is associated with endothelial dysfunction, increased oxidative stress, and enhanced inflammatory responses (22). These processes may exacerbate cerebral ischemia–reperfusion injury (23); second, insulin resistance has been linked to impaired collateral circulation (24), which is a critical determinant of tissue fate in ischemic stroke; And third, patients with higher TyG index may have more severe atherosclerosis and vulnerable plaques (25), potentially leading to more extensive thrombus burden and more challenging revascularization procedures. In addition, insulin resistance reduces endothelial nitric oxide synthase (eNOS) activity and nitric oxide bioavailability, leading to impaired cerebral vasodilation and compromised microvascular perfusion during reperfusion. This endothelial dysfunction manifests as increased blood–brain barrier permeability, enhanced inflammatory cell infiltration, and elevated risk of hemorrhagic transformation following EVT.

Our finding that the TyG index has better predictive value (AUC = 0.714) than either glucose (AUC = 0.618) or TGs (AUC = 0.574) alone or in combination (AUC = 0.633) is particularly noteworthy. This finding supports the concept that the TyG index, as a composite marker of glucose and lipid metabolism, provides more comprehensive information about metabolic dysfunction than individual parameters. Previous studies have reported similar findings in other cardiovascular conditions. For instance, Zhao et al. demonstrated that the TyG index outperformed fasting glucose and TGs in predicting coronary artery calcification (26), while Sánchez et al. reported superior performance of the TyG index in predicting cardiovascular events in patients with type 2 diabetes (27). Our findings align with recent validation studies of metabolic biomarkers in stroke. Lee et al. reported a TyG index AUC of 0.69 for poor outcomes after reperfusion therapy, while Toh et al. demonstrated an AUC of 0.71 for clinical outcomes following intravenous thrombolysis. Our AUC of 0.714 for the TyG index alone and 0.826 for the combined model represents the highest reported discriminative performance for the TyG index in post-EVT outcomes, potentially reflecting our focus on this specific high-risk population and comprehensive covariate adjustment.

The optimal cut-off value of the TyG index in our study was 8.79, with moderate sensitivity (56.9%) but good specificity (75.3%). The relatively high specificity suggests that the TyG index may be particularly useful for identifying patients at low risk of poor outcomes, which could aid in clinical decision-making and resource allocation. The significant improvement in predictive performance when adding TyG index to conventional prognostic factors (AUC from 0.776 to 0.826, *p* = 0.032) underscores its potential clinical utility. This finding is consistent with previous studies showing that incorporation of metabolic parameters enhances the accuracy of outcome prediction in stroke patients (20). The incremental predictive value of the TyG index over established risk factors suggests that it captures additional prognostic information not reflected by traditional clinical and imaging parameters. Our decision to primarily treat the TyG index as a continuous variable in the multivariate analysis was based on statistical best practices, which recommend preserving the continuous nature of variables to maintain statistical power and avoid arbitrary cutoff effects.

Our subgroup analysis revealed that the predictive value of the TyG index was more pronounced in patients aged ≥65 years (AUC = 0.747) compared to younger patients (AUC = 0.643) and in male patients (AUC = 0.726) compared to female patients (AUC = 0.696). The stronger association in elderly patients may be explained by the higher prevalence and severity of insulin resistance with advancing age (28), as well as the potential interaction between age-related changes in cerebrovascular function and metabolic dysfunction (29). The gender difference in the predictive value of the TyG index could be attributed to sexual dimorphism in insulin signaling and glucose metabolism (30), as well as differences in fat distribution and adipokine profiles between men and women (31). In addition, integration with therapeutic interventions such as statin therapy, which has shown promise in improving EVT outcomes (32, 33), may further enhance the clinical utility of TyG index-based risk stratification.

The observed age and sex differences in TyG index prognostic value suggest potential synergistic effects that warrant further investigation. Recent evidence indicates that metabolic markers may demonstrate enhanced predictive capacity in specific demographic subgroups, particularly elderly women, where post-menopausal hormonal changes compound age-related metabolic dysfunction. The interaction between sex and age on insulin resistance patterns may explain why the TyG index performs differently across demographic groups. Future studies with larger sample sizes should examine four-way stratification (elderly women, elderly men, younger women, and younger men) to better understand these complex interactions and optimize risk stratification approaches for different patient populations.

These findings have important clinical implications. First, the TyG index, calculated from routine laboratory tests, represents a readily available and cost-effective prognostic marker that could be easily incorporated into clinical practice. Second, the identification of patients with elevated TyG index may guide more aggressive management strategies, including intensive monitoring, optimization of reperfusion therapy, and targeted metabolic interventions. Third, the stronger predictive value in specific subgroups suggests the potential for more personalized risk stratification and treatment approaches.

Several limitations of our study should be acknowledged. First, as a retrospective study, residual confounding cannot be excluded despite adjusting for multiple covariates. Second, we did not have information on the duration of diabetes or pre-stroke glycemic control, which might influence the interpretation of the admission TyG index. Third, our sample size was determined by convenience sampling rather than prospective power calculation, which may limit the generalizability of our findings. Our derived TyG index cutoff of 8.795 requires external validation in independent cohorts before clinical implementation. Without external validation, the clinical applicability and generalizability of our proposed cutoff remain uncertain. Although post-hoc analysis confirmed adequate power for our primary outcomes, the relatively small sample size, particularly for subgroup analyses, necessitates validation in larger prospective cohorts. Fourth, anthropometric measurements (BMI, WC) were not consistently available, preventing the calculation of derivative TyG indices (TyG-BMI, TyG-WC) that might have enhanced predictive accuracy. Finally, we did not include several well-established prognostic factors for EVT outcomes in our analysis: ASPECTS score, onset-to-groin puncture time, bridging therapy administration, and systematic hemorrhagic transformation assessment. The absence of these variables may have resulted in residual confounding and overestimation of the TyG index’s independent prognostic value. Therefore, in the follow-up work, we will continue to expand the sample size while refining our database collection metrics.

Despite these limitations, our study has several strengths. To our knowledge, this is one of the first studies to specifically investigate the relationship between the TyG index and outcomes after thrombectomy in AIS. The analysis of the incremental value of the TyG index over conventional prognostic factors provides clinically relevant information about its potential utility in risk stratification.

## Conclusion

In conclusion, our study demonstrates that elevated TyG index is independently associated with unfavorable outcomes in patients with AIS after thrombectomy, with superior predictive value compared to individual glucose or TG measurements. The addition of the TyG index to conventional prognostic factors significantly improves outcome prediction, particularly in elderly male patients. These findings suggest that the TyG index could serve as a valuable prognostic marker in clinical practice and may help identify patients who would benefit from more intensive monitoring and management. Future prospective studies with larger sample sizes are warranted to validate our findings and explore whether interventions targeting insulin resistance could improve outcomes in AIS undergoing thrombectomy.

## Data Availability

The original contributions presented in the study are included in the article/supplementary material, further inquiries can be directed to the corresponding authors.
